# Increased Thymic Cell Turnover under Boron Stress May Bypass TLR3/4 Pathway in African Ostrich

**DOI:** 10.1371/journal.pone.0129596

**Published:** 2015-06-08

**Authors:** Hai-bo Huang, Ke Xiao, Shun Lu, Ke-li Yang, Abdur Rahman Ansari, Haseeb Khaliq, Hui Song, Juming Zhong, Hua-zhen Liu, Ke-mei Peng

**Affiliations:** 1 Department of Anatomy, Histology and Embryology, College of Animal Science and Veterinary Medicine, Huazhong Agricultural University, Wuhan, Hubei, People’s Republic of China; 2 Department of Anatomy, Physiology and Pharmacology, College of Veterinary Medicine, Auburn University, Auburn, Alabama, United States of America; University of London, St George's, UNITED KINGDOM

## Abstract

Previous studies revealed that thymus is a targeted immune organ in malnutrition, and high-boron stress is harmful for immune organs. African ostrich is the living fossil of ancient birds and the food animals in modern life. There is no report about the effect of boron intake on thymus of ostrich. The purpose of present study was to evaluate the effect of excessive boron stress on ostrich thymus and the potential role of TLR3/4 signals in this process. Histological analysis demonstrated that long-term boron stress (640 mg/L for 90 days) did not disrupt ostrich thymic structure during postnatal development. However, the numbers of apoptotic cells showed an increased tendency, and the expression of autophagy and proliferation markers increased significantly in ostrich thymus after boron treatment. Next, we examined the expression of TLR3 and TLR4 with their downstream molecular in thymus under boron stress. Since ostrich genome was not available when we started the research, we first cloned ostrich TLR3 TLR4 cDNA from thymus. Ostrich TLR4 was close to white-throated Tinamou. Whole avian TLR4 codons were under purify selection during evolution, whereas 80 codons were under positive selection. TLR3 and TLR4 were expressed in ostrich thymus and bursa of fabricius as was revealed by quantitative real-time PCR (qRT-PCR). TLR4 expression increased with age but significantly decreased after boron treatment, whereas TLR3 expression showed the similar tendency. Their downstream molecular factors (IRF1, JNK, ERK, p38, IL-6 and IFN) did not change significantly in thymus, except that p100 was significantly increased under boron stress when analyzed by qRT-PCR or western blot. Taken together, these results suggest that ostrich thymus developed resistance against long-term excessive boron stress, possibly by accelerating intrathymic cell death and proliferation, which may bypass the TLR3/4 pathway. In addition, attenuated TLRs activity may explain the reduced inflammatory response to pathogens under boron stress.

## Introduction

Thymus is the primary immune organ for T cell development in jawed vertebrates [1]. Normal thymus maintains distinct architecture including outer compact cortex where T cell precursors commit to T cell lineage and gain the ability to interact with self-antigen and central loose medulla where most autoreactive thymocytes are deleted. During T cell development in thymus, only 15% of thymocytes are mature and leave the organ to form the majority of the peripheral T cells [2]. The balance of apoptosis, autophagy and proliferation plays a major role in thymic cell turnover, and the imbalance of these processes is associated with the degeneration of thymus under stress or with age [2–5]. Furthermore, multiple environmental stressors, such as pathogenic infection and imbalance of trace elements, can disrupt normal thymus organization and reduce organ size by acute depletion of immature CD4+CD8+ thymocytes in cortex, which are associated with impaired peripheral immune response [2, 6].

The African ostrich (*Struthio camelus*) is a terrestrial flightless giant bird and regarded as the living fossil of primitive birds, as it could be a good model for evolutionary research of ancient avians [7]. Furthermore, ostrich breeding as food animals is becoming more common in Europe and Asia due to its commercial value with low fat and low cholesterol in ostrich meat [8, 9].

Boron in nutritional amounts is suggested to be beneficial for the performance of diverse tissues, such as bone, nerve tissue and muscle, and has been used for ostriches [10–12]. Nevertheless, excessive boron (> 400 mg/kg) has been reported to inhibit the development of multiple immune organs (eg, thymus, spleen) in chickens and rats [6, 13]. However, up to date, no report has studied the effects of excessive boron stress on ostrich thymus and the related mechanism.

Toll like receptors (TLRs) are major components of innate immune system in that they can recognize multiple endogenous and exogenous stress signals and induce an immune response [14]. TLRs are expressed in the thymus of chickens and are suggested to participate in thymus development by inducing downstream factors [15, 16]. Furthermore, abnormal thymic development is associated with increased expression of TLR4 [17], aberrant activation of transcription regulators (NF-κB, MAPK) [18, 19], and overexpression of proinflammatory cytokines (IL-6 and IFN) [20, 21].

Though there is no direct evidence that boron derived compound can be recognized by TLRs, boron stress can increase intracellular stress signals, such as the subunit of heat shock proteins [22], which are the ligands of TLR4. TLR3 can also recognize the host mRNA [14], which may be released from necrotic tissue. Therefore, we hypothesized that TLRs may participate in the process of boron stress in thymus. To this end, ostrich TLR3 and TLR4 were cloned firstly, since the ostrich genome was not available at the time when the project was started. Next, the impact of chronical boron stress on thymus was assessed, and the expressions of TLR3 and TLR4 with their downstream factors were also evaluated.

## Materials and Methods

### Ethics Statement

This study was approved by the ethics committee of Huazhong Agricultural University (approval permit number 31272517). Protocols for animal were performed according to pertinent guidelines (No. 5 Proclaimation of the Standing Committee of Hubei People’s Congress, P.R. China). Exsanguination and decollation were used for humane form of euthanasia. All efforts were made to minimize animal suffering.

### Animals

Healthy 1-day-old African ostriches (*Struthio camelus*) were obtained from Jinlu Ostrich Co., Ltd. (Henan Province, China). Animals were randomly divided into 3 groups (45d, 90d, and 90dB; *n* = 3), and housed under conventional conditions without any vaccinations. Group 45d and 90d received distilled water while Group 90dB received natrium biboricum through drinking water at 640 mg/L dose daily which is known to be excessive and toxic for immune related organs [6, 13]. The thymuses were collected at 45 days (Group 45d), and 90 days (Group 90d and 90dB). A portion of the thymuses was fixed in 4% paraformaldehyde, and the rest portion was frozen in liquid nitrogen. Ostriches were monitored daily, and no unexpected death was observed.

### Special staining and immunochemistry

Tissues were embedded in paraffin wax and sectioned at 4 μm and mounted on polylysine-coated slides. Masson’s trichrome stain [23] was applied to tissue sections to show the changes of basic histological structures after excessive boron treatment. For immunochemistry, the antibodies of TLR4 (1:50), LC3A/B (1:50), PCNA (1:100), PCK (1:50) and S100 (1:50) were used, which either target the amino acid sequences sharing high identity (>70%) with those found in ostriches, or their applicability for birds are declared by each company (S1 Table). The immunochemistry staining was performed as described [16]. For apoptotic staining, a mouse IgM anti-ssDNA monoclonal antibody (1:10; EMD Millipore, Billerica, USA) was used. The procedure for this antibody was the same as mentioned above except that the sections were treated with 0.1 mg/mL saponin and 20 μg/mL proteinase K in PBS for 20 min at 37°C, incubated in 50% (v/v) formamide in distilled water for 20 min at 56°C and cooled in cold PBS for 5 min, instead of heat induced antigen retrieval in a microwave oven, and employed anti-mouse IgM SABC kit (Boster, Wuhan, China) instead of other secondary antibodies kit. Chromotrope 2R stain [24] was performed after TLR3 and TLR4 immunostaining to visualize eosinophils.

The expressions of TLR4, LC3A/B and PCNA were measured using integrated optical density (IOD) ratio as described [16], and the total numbers of ssDNA positive cell per 1 × 10^5^ μm^2^ in thymic cortex were quantified.

### Cloning of ostrich TLR3 and TLR4 cDNA

Total RNA was isolated from thymus using TRIzol reagent (TaKaRa). First strand cDNA was synthesized using the RevertAid First Strand cDNA Synthesis Kit (Thermo Scientific) and DNase I (Thermo Scientific). Degenerative PCR primers for ostrich TLR4 and TLR3 were designed based on the multiple alignments of previously reported sequences in birds (S2 Table). The first PCR reactions were performed with the degenerative primers (Table 1) to obtain the partial sequences of ostrich TLR3 and TLR4. Next, nested primers for ostrich TLR4 (Table 1) were designed from the obtained sequence to perform 5’ and 3’RACE using 5’ RACE System for Rapid Amplification of cDNA Ends (Invitrogen, USA) and Smart RACE cDNA amplification kit (Clontech, USA), respectively. The RACE products were ligated into pMD18-T vector, sequenced and assembled to obtain the full-length ostrich TLR4 cDNA.

**Table 1:** The primers used in this study.

Name	Sequence (5’→3’)	Purpose
TLR3-SC-DP-F	TGGMMHTTTCTCCYTCRCCTTTTC	PCR
TLR3-SC-DP-R	AADGCKAYYCTCCACCCTTCAAA	PCR
TLR4-SC-DP-F	CTTTCCASCTTRRTTYTAACTGSCAAT	PCR
TLR4-SC-DP-R	CATYYGYACCTGRCAVAGTCCA	PCR
TLR4-SC-GSP1	CCTTGGTGAGCTGGCTTTAAGGTCC	3'-RACE
TLR4-SC-GSP2	GTTGGAGAATTCAGTGACATTCAGA	3'-RACE
TLR4-SC-GSP1	GGGCAGGTCAGTCAGAGAAG	5'-RACE
TLR4-SC-GSP2	AGCTGCTGGCCCCAGGTAGT	5'-RACE
TLR4-SC-For	TCGGCTCTCCTTCACCAAAT	qRT-PCR
TLR4-SC-Rev	GGTAGGAGCCAGGACCAATT	qRT-PCR
TLR3-SC-For	TGCAACACCAGACTACCATG	qRT-PCR
TLR3-SC-Rev	CTTGGTTTGCTGGTTGGCTT	qRT-PCR
p100-SC-For	CCATCGTCGTCAGAAGCAAG	qRT-PCR
p100-SC-Rev	GTGGGATCAGAGTCCAAGCT	qRT-PCR
IRF1-SC-For	ACCCTGACCCTAAAACCTGG	qRT-PCR
IRF1-SC-Rev	GCAGCATTCGGTACACTCTG	qRT-PCR
IL6-SC-For	CTCAGCTACCCAGAAGTCCC	qRT-PCR
IL6-SC-Rev	CGGAGGATAAGGTGTGTGGT	qRT-PCR
IFN-SC-For	GAAGGTAGTAAGCAACGCCTCCT	qRT-PCR
IFN-SC-Rev	CACAGCATGTACAGTGGTCATCA	qRT-PCR
Actin-SC-For	TGGTCGTGACCTGACTGATT	qRT-PCR
Actin-SC-Rev	TCACGGACAATCTCACGTTC	qRT-PCR

The qRT-PCR primers for TLR3, TLR4 and actin were designed from our cloning results (KM651767, KM408431 and KJ729106.1), since the ostrich genome was not available when we started the research. The qRT-PCR primers for p100, IRF1, IL6 and IFN were designed from subsequent ostrich genomic information (KFV79432.1, KFV73539.1, KFV82123.1 and KFV87588.1).

### Sequence analysis of ostrich TLR4

Amino acid sequences were deduced using the NCBI ORF finder program. The protein domains were predicted using the SignalP 4.1, TMPRED and SMART programs. The genomic structure of TLR4 gene was deduced from mRNA and DNA sequence using NCBI SPIDEY program. For adaptive evolution analysis, the 36 known TLR4 sequences of bird were retrieved from GenBank (S3 and S4 Tables), and the ostrich TLR4 was from our cloning result. Sequences were aligned by MUSCLE, analyzed using the single likelihood ancestor counting (SLAC) method (α = 0.1), random-effects likelihood (REL) method (Bayes factor > 50), fixed effects likelihood (FEL) method (α = 0.1), and mixed-effects model of evolution (MEME) method (α = 0.1) on the DATAMONKEY webserver. The normalized dN-dS observed at each residue were plotted using the Prism software 5.01(GraphPad Software, Inc., San Diego, USA). Phylogenetic analysis of the TLR4 proteins in birds, reptiles, mammals and fish (S3 and S4 Tables) was carried out by the maximum likelihood method using MEGA 6.0 program, and the reliability of the estimated tree was evaluated by the bootstrap method with 1000 pseudo-replications.

### Quantitative real time PCR

Expression patterns of TLRs and related genes in ostrich tissues were analyzed based on quantitative real time PCR (qRT-PCR) using β-actin as the reference gene. All the primer sequences, TLR3, TLR4, P100, IRF1, IL-6, IFN and β-actin were described in Table 1. Real-time PCR was performed using a Bio-Rad CFX Connect real-time PCR detection system (Bio-Rad, Hercules, CA)with SYBR Select Master Mix for CFX (Bio-Rad, Hercules, CA). Real-time PCR conditions were as following: pre-denaturation at 95°C for 30 min, followed by 45 cycles of 95°C for 10 s, annealing at 55°C for 20 s, and elongation at 72°C for 20 s. PCR was performed in triplicate, gene expression levels were quantified using the Ct (ΔΔCt) value method.

### Western blot

Proteins were homogenized in lysis buffer containing a protease inhibitor. Protein concentration was measured by the Bradford method using BSA as standard. Samples of 40 μg of total protein were separated by SDS–PAGE and electrotransferred to a PVDF membrane (Millipore, Bedford, USA). Immunodetection was performed using antibodies against TLR4 (1:500) from Boster (Wuhan, China), JNK (1:500) and Actin (1:1000) from Santa Cruz Biotechnology (CA, USA), ERK (1:1000) from Bioworld (MN, USA), P38 (1:1000) from Epitomics (CA, USA), and the reliability of these antibodies were provided in S1 Table.

### Statistical analysis

Data were presented as mean ± SEM,with samples derived from 3 individuals in each group. Significant differences between groups were studied using a Student’s *t*-test. All analyses and graphic representations were performed with Prism software 5.01(GraphPad Software, Inc., San Diego, USA). Data trends are marked with one asterisks (*) for *P* < 0.1 and two asterisks (**) for *P* < 0.05 respectively. *P* < 0.05 was considered significant.

## Results

### Ostrich thymic structure was maintained under boron stress

**Fig 1:**
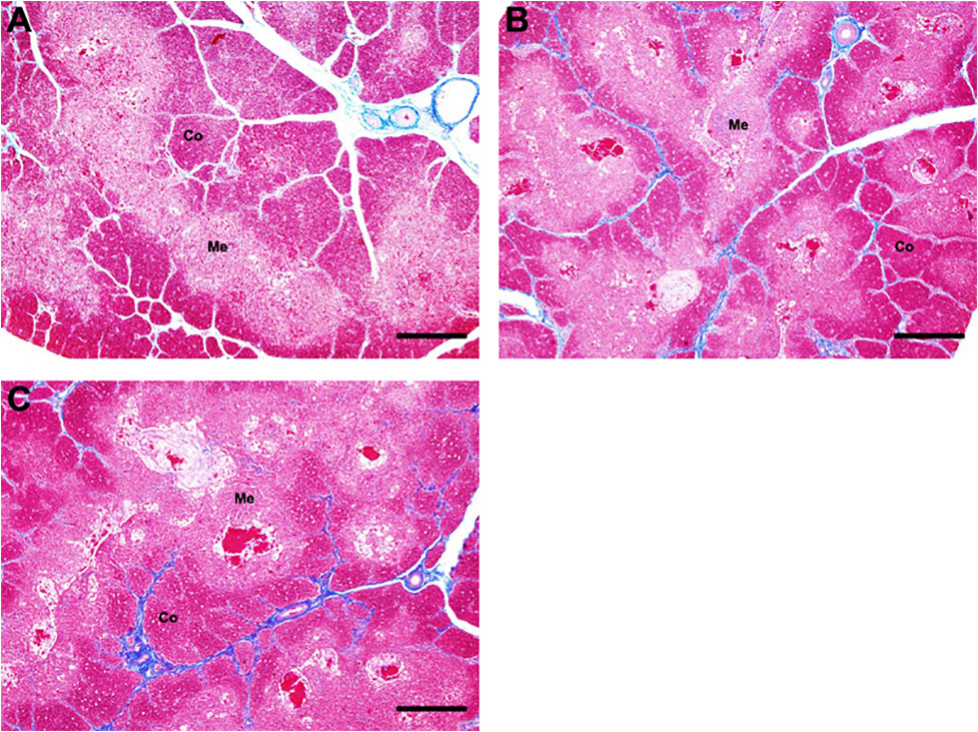
The changes of histological structure of ostrich thymus in different groups (Masson’s staining). **(A)** Thymus in group 45d; **(B)** Thymus in group 90d; **(C)** Thymus in 90dB. Co, cortex; Me, medulla. Bars, 500 μm.

In previous studies a toxic effect of boron disrupting the immune organs’ structures was shown in other animals when the concentration was more than 400 mg/L [6, 13]. Here, we tested the possible effect of boron stress on thymic structure after 90-days of continuous boron intake (640 mg/L). Surprisingly, no obvious change was observed in ostrich thymic interstitial—parenchymal, and cortico-medullary structure among different groups (Fig 1A, 1B and 1C).

### Boron stress increased thymic apoptosis in ostriches

**Fig 2:**
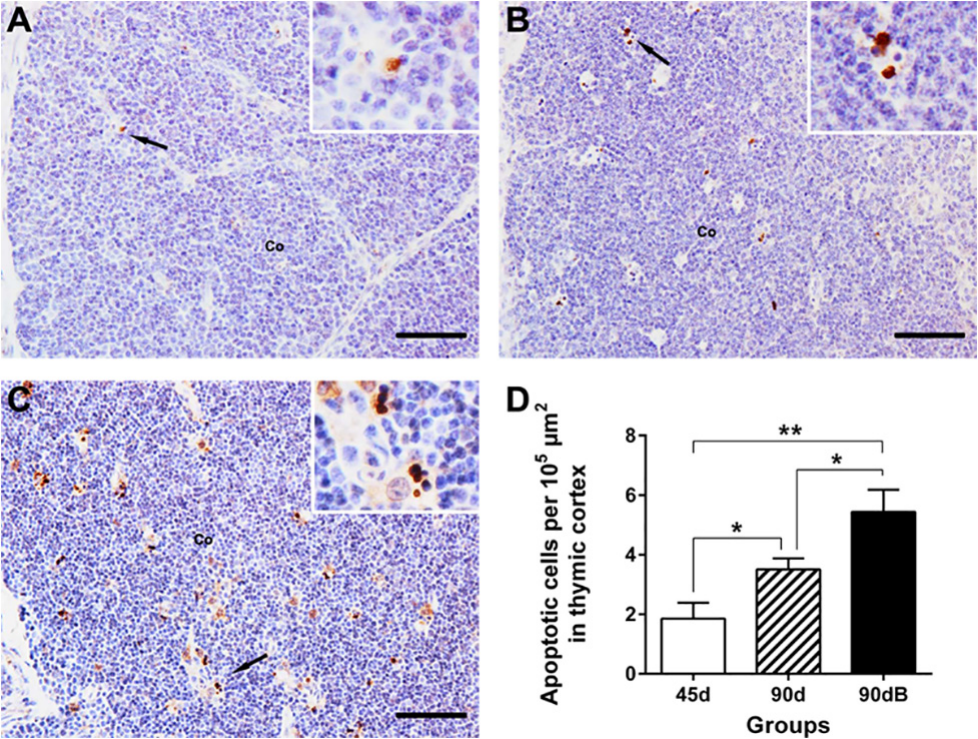
Changes of the apoptotic cell numbers in ostrich thymic cortex from different groups using anti-ssDNA mAb. (**A)** in group 45d; (**B)** in group 90d; (**C)** in 90dB. Co, cortex. Bars, 50 μm. (**D)** Statistic analysis of numbers of apoptotic cells per 10^5^ μm in thymus cortex. Data are representative of at least five tissue sections per ostrich (*n* = 3 per group). *p < 0.10, **p < 0.05.

Apoptosis is one of the important ways to maintain intrathymic homeostasis, therefore we analyzed it in ostrich thymus, using specific antibodies against ssDNA [25]. In normal developing ostrich thymus, the apoptotic cells mainly distributed in thymic cortex and their number increased with age (*P* < 0.1) (Fig 2A, 2B and 2D). In boron-stressed thymus, thymic apoptosis cells displayed an increased tendency (*P* < 0.1) (Fig 2B, 2C and 2D) compared with that of normal thymus at day 90.

### Boron stress increased thymic autophagy in ostriches

**Fig 3:**
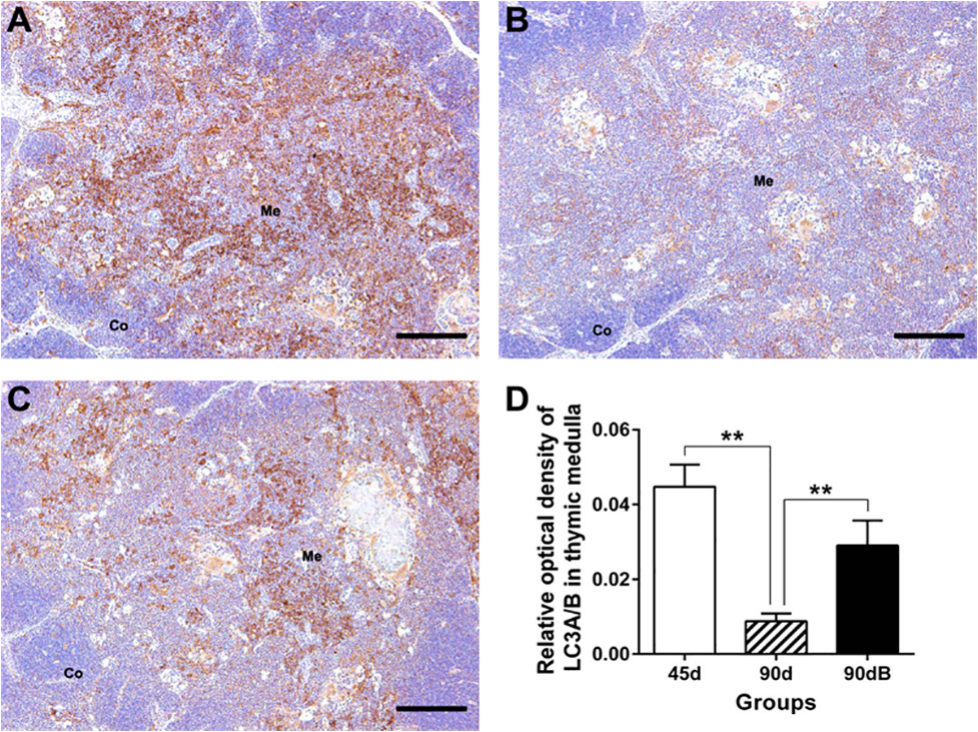
Expression of LC3A/B in ostrich thymic medulla from different groups using related mAb. (**A**) in group 45d; (**B**) in group 90d; (**C**) in 90dB. Me, medulla. Bars, 200 μm. (**D**) Statistic analysis of protein expression of LC3A/B (IOD) in thymus medulla. Data are representative of at least five tissue sections per ostrich (*n* = 3 per group). **p < 0.05.

Recent studies also showed that autophagy played a critical role for T cell development [4], thus we evaluated the expression of LC3A/B, the classic index for autophagy [3], in ostrich thymus. The LC3A/B was mainly located in thymic medulla. Its protein expression decreased significantly with age (*P* < 0.05) in normal ostrich developing thymus (Fig 3A, 3B and 3D), but significantly increased (*P* < 0.05) (Fig 3B, 3C and 3D) in boron-stressed thymus, compared with that of normal thymus at day 90.

### Boron stress increased thymic proliferation in ostriches

**Fig 4:**
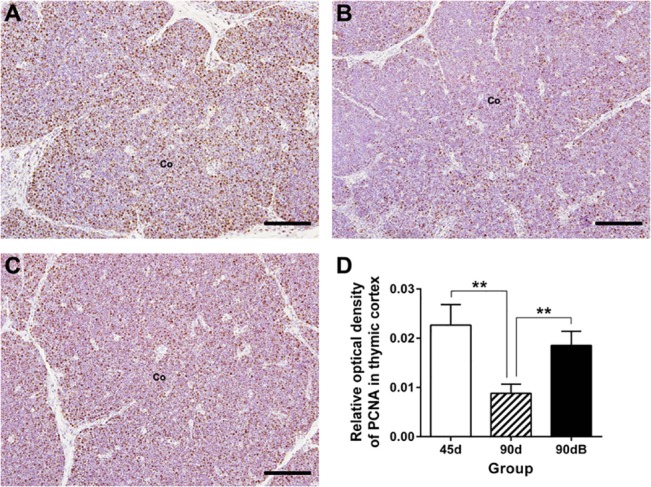
Expression of PCNA in ostrich thymic cortex from different groups using related mAb. (**A)** in group 45d; (**B)** in group 90d; (**C)** in 90dB. Co, cortex. Bars, 100 μm. (**D)** Statistic analysis of protein expression of PCNA (IOD) in thymus cortex. Data are representative of at least five tissue sections per ostrich (*n* = 3 per group). **p < 0.05.

To determine the reverse side of thymic clearance, we studied thymic proliferation using a specific antibody against PCNA. In normal developing thymus of ostrich, PCNA was mostly expressed in thymic cortex, and its expression decreased significantly with age (*P* < 0.05) (Fig 4A, 4B and 4D). Under boron stress, the protein level of PCNA increased significantly (*P* < 0.05) (Fig 4B, 4C and 4D).

### Character of ostrich *TLR3* and *TLR4*

Considering the possibility of TLR signals in participating ostrich thymic boron stress, we analyzed the expression of TLR3 and TLR4, the two classic ones of TLRs. TLR3 can recognize both host mRNA and viral double stranded RNA, whereas TLR4 can recognize bacterial lipopolysaccharide and endogenous heat shock proteins [14]. At the time the study began, there were no reports pertaining to TLRs in the African ostrich, so we first had to identify the partial sequence of ostrich TLR3 and TLR4.

**Fig 5:**
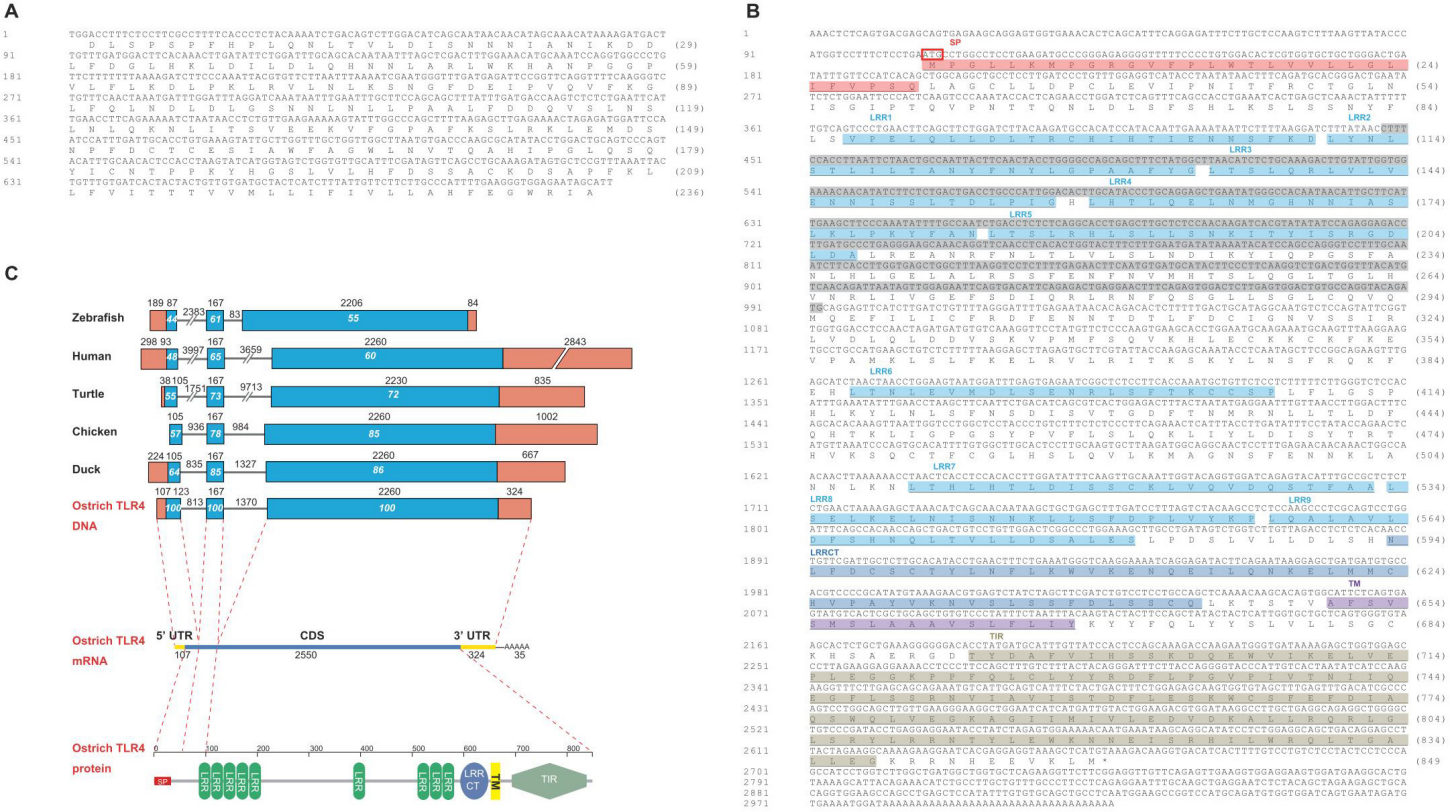
TLR3 and TLR4 cDNA sequence in African ostrich (*Struthio camelus*). (**A**) The partial TLR3. (**B**) The complete TLR4. The sequence marked with gray in panel **B** indicated the partial TLR4 sequence cloned by us at first. The start codon is marked with a red rectangle and the stop codon is marked with an asterisk (*); The SP domain is marked with pink color, the LRRs domains with blue color, the LRRCT domain with deep blue color, the TMD domain with purple color and the TIR with brown. (**C**) Schematic representation of the TLR4 genes in birds, reptiles, mammals and fish. The ostrich genomic sequence of TLR4 was deduced from cloning TLR4 mRNA (KM408431) and ostrich genomic scaffold (KL205999.1) provided by BGI, and the others are retrieved from NCBI and described in S4 Table. Exons are indicated by boxes, coding regions by blue boxes, non-coding regions by orange boxes. The numbers above the organizations represent the sizes (bp) of corresponding regions, and the ones inside the boxes represent the identity (%) of corresponding sequences compared with those of ostrich. In the structure of ostrich TLR4 mRNA, the coding sequences (CDS) are indicated with blue line, the 5’ untranslated region (5’UTR) and 3’ untranslated region (3’UTR) are indicated with yellow line. SP signal peptide, LRRs leucine-rich repeats, LRRCT leucine-rich repeat C-terminal domain, TM transmembrane domain, TIR Toll/interleukin-1 receptor.

The partial TLR3 (KM651767) and TLR4 sequence are 712 bp and 546 bp, encoding 236 and 182 amino acids (aa) respectively (Fig 5A and 5B). These two partial amino acids sequences share 91% and 82% identity with those of the duck (XP_005009038.1; AER24529.1) respectively, and share both 99% identity with those from succeeding ostrich genome (XP_009674996.1; XP_009666473.1) (Table 2), which confirmed the reliability of our cloning results. Since TLR4 is the first identified TLR in mammals and also the common model for TLRs research, we cloned the full length of ostrich TLR4 by RACE, to further analyze ostrich TLRs gene character. The complete ostrich TLR4 cDNA (KM408431) is 3016 bp, including an ORF of 2550 bp encoding a polypeptide of 849 amino acids (Fig 5B). The deduced amino acid sequence consists of a signal peptide, LRR domain, transmembrane domain and TIR domain (Fig 5B), suggested a typical structure of TLR family proteins [26].

**Table 2:** Sequence identity (%) and similarity (%) between ostrich TLRs proteins from molecular cloning and those of ostrich, duck, chicken, turtle, human and zebrafish retrieved from GenBank.

Species	TLR3 (partial)		TLR4 (partial)		TLR4 (full)	
	Identity (%)	Similarity (%)	Identity (%)	Similarity (%)	Identity (%)	Similarity (%)
Ostrich	99	99	99	99	99	99
Duck	91	95	82	87	80	88
Chicken	87	93	80	89	79	88
Turtle	81	91	64	78	65	78
Human	70	84	53	67	47	64
Zebrafish	57	71	39	55	38	56

The proteins ID from GenBank are as follows: duck TLR3 XP_005009038.1; chicken TLR3 NP_001011691.3; turtle TLR3 XP_006128521.1; human TLR3 NP_003256.1; zebrafish TLR3 NP_001013287.2; ostrich TLR3 XP_009674996.1; duck TLR4 AER24529.1; chicken TLR4 NP_001025864.1; turtle TLR4 NP_001273862.1; human TLR4 NP_612564.1; zebrafish TLR4 NP_997978.1; ostrich TLR4 XP_009666473.1. The proteins for ostrich TLR 3 (KM651767) and TLR4 (KM408431) deduced from our cloning result were indicated in Fig 5A and 5B.

Since the ostrich genome was available after we cloned ostrich TLRs, we adopted ostrich genomic scaffold (KL205999.1) from GenBank and our cloning result to deduce the genomic sequence of ostrich TLR4. The ostrich genomic TLR4 is 5164 bp, consists 2 introns (813 and 1370 bp) and 3 exons (230, 167 and 2584 bp), the same as that of duck, chicken, turtle, human and zebrafish (Fig 5C). The coding regions of exon 1 and 3,and exon 2 of ostrich TLR4 gene share 44~64%, 55~86% and 61~85% identity compared those of other species, this suggested a conservative genomic structure of TLR4 in various species.

### Polygenetic and evolution analysis based on ostrich *TLR4*

**Fig 6:**
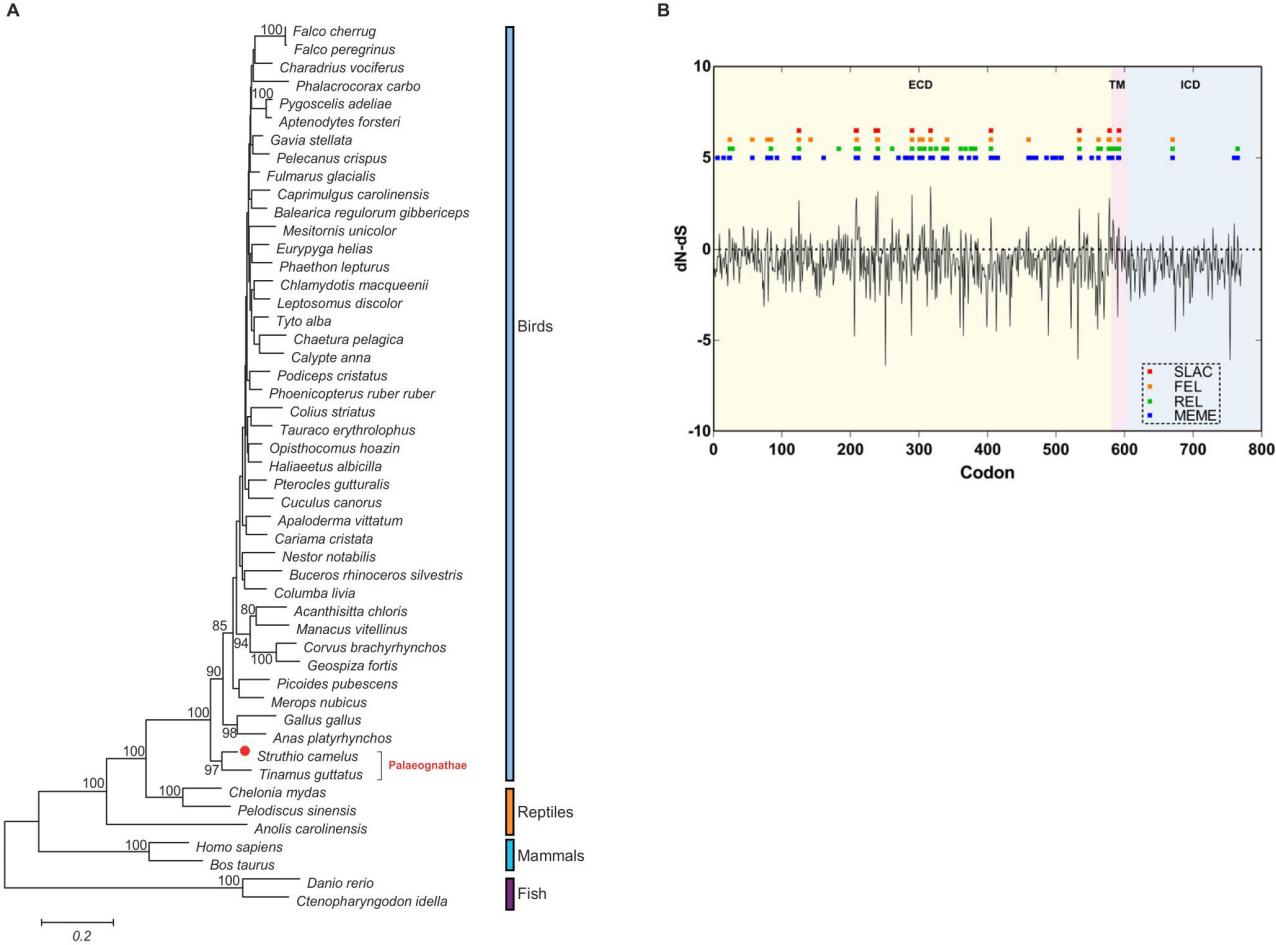
Polygenetic and evolutionary analysis of TLR4 in different species. (**A**) Polygenetic tree constructed from the amino acid sequences of TLR4 molecule in 49 species. Amino acid sequences were aligned using MUSCLE and tree was constructed by the maximum likelihood method supported with 1000 bootstrap replications using MEGA6 software. Boot strap values are indicated above the lines, and the scale bar corresponded to the evolutionary distance. The adopted sequences IDs for polygenetic analysis are stated in S3 and S4 Tables. (**B**) Map of nucleotide substitution patterns at TLR4 loci in 37 birds. The *x*-axis represents the codon loci aligned with that of ostrich. The black line represents the value of normalized dN-dS calculated in SLAC at each site. The blue, green, orange and red boxes represent the sites under positive selection detected respectively by MEME, REL, FEL and SLAC (DATAMONKEY). ECD extracellular domain; TM transmembrane domain; ICD intracellular domain.

Phylogenetic trees revealed that TLR4 sequences from birds were more closely related to reptiles than those of mammals and fish (Fig 6A). In addition, TLR4 of ostrich (*Struthio camelus*) was more closely related to that of white-throated Tinamou (*Tinamus guttatus*), both of which belong to the superorder Palaeognathae. This result was in accordance with conventional taxonomy of birds.

Previous studies revealed an episodic positive selection of TLR4 (only 270 codons in ECD) in 18 avian species [27]. Since then, the amount and quality of available TLR4 data has increased dramatically in a short period of time (S3 and S4 Tables). Therefore, we performed the adaptive evolution analysis of TLR4 (771 codons in ECD, TM and ICD) in 37 birds, which contain the annotated TLR4 mRNA sequences. The dN/dS was 0.434 using SLAC method (DATAMONKEY), confirmed a purifying selection acting on all the TLR4 protein. Positive selection was also observed across all loci using SLAC, REL, FEL and MEME method (DATAMONKEY) [28] (Fig 6B). There are 80 sites under the positive selection (90% in ECD, 6% in TM and 4% in ICD) which were detected by at least one method of them, and 15 key sites under the positive selection (87% in ECD, 7% in TM and 7% in ICD) which were detected by at least three method of them (Fig 6B, Table 3).

**Table 3:** The numbers (and percentage) of positively selected sites of ostrich TLR4.

TLR4 domains	SLAC	FEL	REL	MEME	Either (>1) *	Either*(>3) *
**ECD**	10 (91%)	20 (91%)	27 (84%)	54 (92%)	72 (90%)	13 (87%)
**TM**	1 (9%)	1 (5%)	3 (9%)	2 (3%)	5 (6%)	1 (7%)
**ICD**	0 (0%)	1 (5%)	2 (6%)	3 (5%)	3 (4%)	1 (7%)

ECD, extracellular domain; TM, transmembrane domain; ICD, intracellular domain.

*” Either (>1)” and” Either (>3)” indicate the sites which were detected using at least one or three methods of SLAC, FEL, REL and MEME.

### TLR3 and TLR4 were expressed in ostrich central immune organs

**Fig 7:**
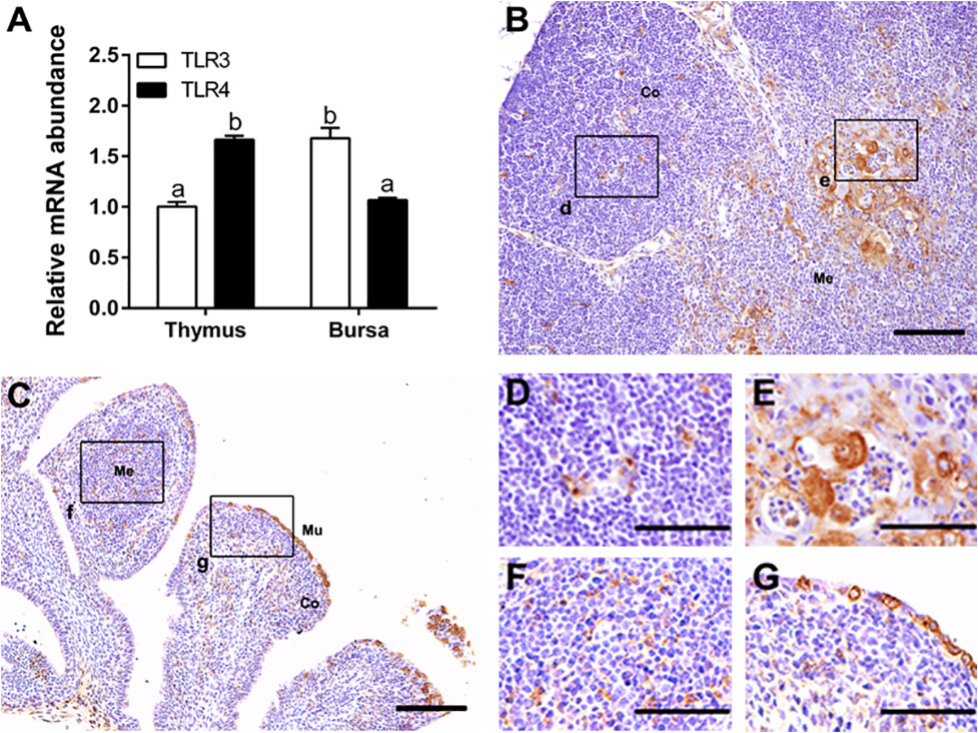
Expression of TLR3 and TLR4 in ostrich thymus and bursa of fabricius. (**A**) The quantification of TLR3 and TLR4 mRNA in ostrich thymus and bursa of fabricius at day 45 (*n* = 3) using real-time PCR. Vertical bars represent the mean±SEM, and bars with different letters mean significantly different (*P* < 0.05). (**B)** In ostrich thymus, TLR4-positive staining was mainly found in medulla and cortico-medullary junction (**E**), and a few in cortex (**D**). (**C**) In ostrich bursa of fabricius, TLR4-positive staining was observed in some mucosal epithelium (**G**) and medulla (**F**). The black rectangle in panels **B** and **C** is enlarged in right bottom of those panels. Co, cortex; Me, medulla; Mu, mucosal epithelium. Bars, 100 μm (**B** and **C**), 50 μm (**D**—**F**).

Our previous study revealed the constitutive expression of TLR4 in chicken thymic epithelial cells [16]. There was no report about the expression of TLRs in ostrich’s central immune organs. Therefore, we assessed the transcript of TLR3 and TLR4 in ostrich thymus and bursa of fabricius using qRT- PCR. Both TLR3 and TLR4 mRNA was detected in ostrich central immune organs, but the expression level was distinctly different. High level of TLR4 transcript was detected in thymus, low in bursa of fabricius, whereas the level of TLR3 transcript was higher in bursa of fabricius than thymus (*P <*0.05) (Fig 7A).

The tissue distribution of TLR4 in ostrich thymus and bursa of fabricius was studied subsequently, since there are available antibodies against human TLR4 which has been used to identify avian TLR4 [14]. In ostrich thymus, TLR4 was mainly distributed in medulla and cortico-medullary junction (Fig 7B and 7E), and lower level in the cortex (Fig 7B and 7D). In bursa of fabricius, TLR4 was mainly located in mucosal epithelium, (Fig 7C and 7G) and in medulla (Fig 7C and 7F).

Furthermore, we adopted the reliable avian antibodies against PCK [16] and S100 [29] to analyze the colocalization of TLR4 and thymic stromal cells and found that TLR4 was distributed in similar areas as PCK and S100 in ostrich thymus (Figures A-D in S1 File). We also performed the first double staining of eosinophils (by special dye) and TLR4 (by antibody) in the same section of ostrich thymus, and found no overlap of these two signals (Figure E in S1 File). Together, ostrich TLR4 was distributed both on thymic PCK positive and S100 positive stromal cells but not in thymic eosinophils.

### Expression of TLR3 and 4 were attenuated under boron stress

**Fig 8:**
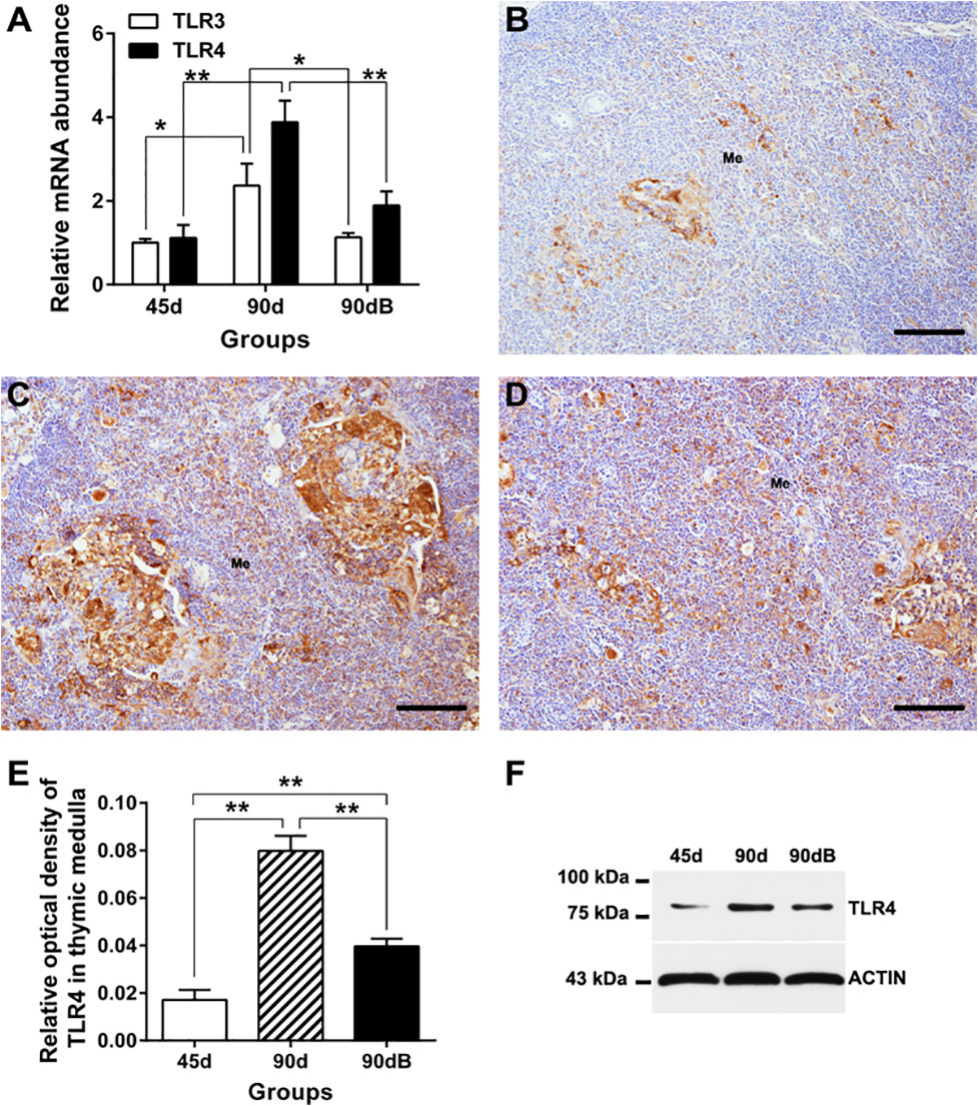
Expression profiles of ostrich TLR3 and TLR4 in thymus from different groups. (**A)** Quantitative real-time PCR analysis of TLR3 and TLR4 mRNA in thymus from the three groups. (**B–D)** Expression of TLR4 protein in group 45d (**A**), 90d (**B**) and 90dB (**D**). Bars, 100 μm. (**E)** Histological analysis of protein expression of TLR4 (IOD) in thymus medulla. Data are representative of at least five tissue sections per ostrich (*n* = 3 per group). **p < 0.05. (**F)** Western blotting analysis of TLR4 protein in ostrich thymus.

To study the role of TLRs signals under boron stress, we analyzed the expression of TLR3 and TLR4. In normal thymus, TLR4 mRNA and protein increased significantly with age (*P* < 0.05) (Fig 8A, 8B, 8C, 8E and 8F), whereas TLR3 mRNA also showed an increased tendency (*P* < 0.1) (Fig 8A). Unexpectedly, TLR4 mRNA and protein decreased significantly (*P* < 0.05) (Fig 8A, 8C, 8D, 8E and 8F), and TLR3 mRNA displayed a decreased tendency (*P* < 0.1) (Fig 8A) in boron-stressed thymus, compared to those in normal developing thymus at day 90.

### Expression of the downstream genes of TLRs under boron stress

**Fig 9:**
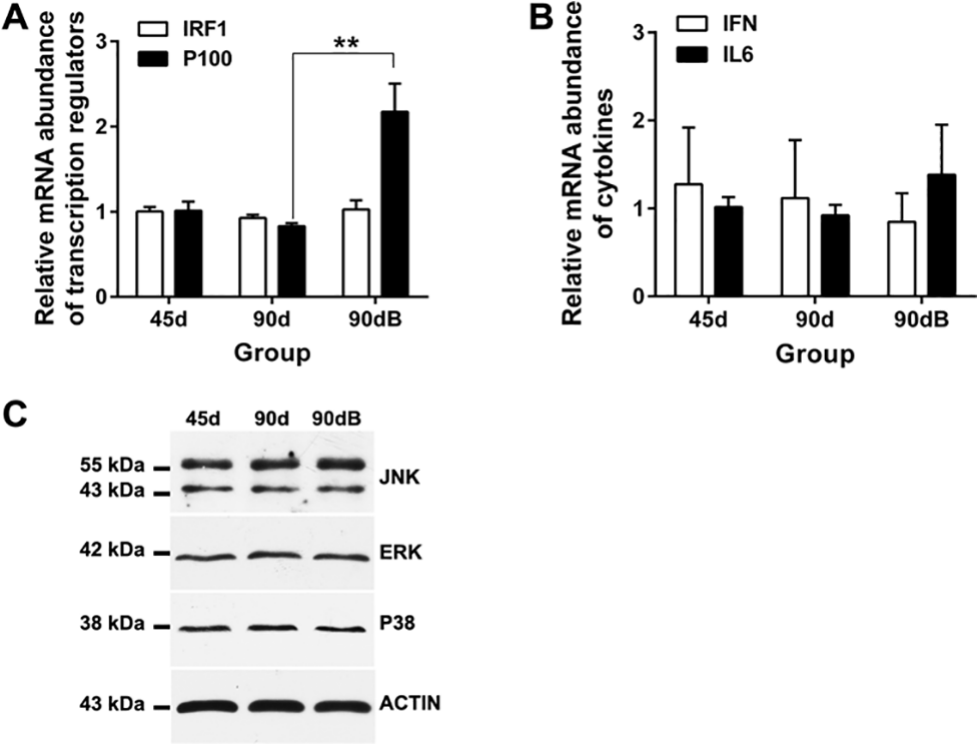
Expression profiles of ostrich TLRs’ downstream products in thymus from different groups. (**A**) qRT-PCR analysis of IRF1 and p100 in ostrich thymus. (**B**) qRT-PCR analysis of IFN and IL-6 in ostrich thymus. **p < 0.05. (**C**) Western blotting analysis of JNK, ERK and p38 protein in ostrich thymus.

Further to study the activity of downstream products of TLRs under boron stress, we analyzed the gene expression of IRF1, p100, IFN and IL-6. Their mRNA levels did not change significantly (Fig 9A and 9B) with age in normal developing thymus of ostrich, or after boron treatment, except that the transcriptional regulator (p100) increased significantly (*P* < 0.05) after boron treatment (Fig 9B). Western blotting analysis of the proinflammatory transcriptional regulators (JNK, ERK and p38) also showed no obvious change among different groups (Fig 9C).

## Discussion

Thymus is a common target for nutritional disorder [2, 6], and excessive boron supplement was suggested to be harmful for immune organs [6, 13]. In this study, we investigated the impact of long-term intake of excessive boron on ostrich thymus and the role of TLR3 and TLR4 with their downstream products in this process for the first time.

Unlike the rat spleen (640 mg/L water daily for 60 days) [13], ostrich thymus maintained an integrated tissue structure under excessive boron stress (640 mg/L water daily for 90 days). This observation may be due to difference in animal species with the different resistant against boron stress and the subsequent recovery mechanism following long-term boron stress, since the previous study indicated that the toxic effect of excessive boron on chicken thymus was relieved following the first 3 weeks [6]. Thus, ostrich thymus may adapt to the boron stress better than other animals after long-term treatment of excessive boron.

Apoptosis, autophagy and proliferation are the classic process to determine the fate of thymic cells. Increasing apoptosis and defective autophagy are associated with the abnormal development of thymus [4, 30], and down-regulation of proteins related to proliferation, including PCNA and CDK2, was observed in degenerated thymus of old individuals [5]. In normal developing thymus, thymic apoptotic cells showed an increased tendency, and thymic autophagy related protein- LC3A/B and proliferation related protein—PCNA decreased significantly with age. Their trends are similar to previous studies in mammals [3, 5]. The increasing cell death and decreasing cell proliferation in thymus of older individuals may undercut the production of T cells. In fact, the output of T cells from thymus did decrease with age [31]. After long-term treatment of excessive boron, the numbers of apoptotic cell were further increased, and the expressions of LC3A/B and PCNA in ostrich thymus were increased significantly, compared with those of normal thymus at the same age. Previous studies suggested a reciprocal relationship between apoptosis and autophagy, since the inhibition of apoptosis can induce autophagy, and the inhibition of autophagy can induce apoptosis [32]. The cell proliferation is also suggested to be inversely related to cell apoptosis [33]. Up-regulated expression of apoptosis, autophagy and proliferation in ostrich thymus may imply a complex relationship among them. Taken together, increasing thymic apoptosis, autophagy and proliferation reflected an accelerating cell turnover and may accomplish a new balance, which help thymus adapt the long-term excessive boron stress.

The phylogenetic tree suggested that theTLR4 sequence was conserved among species and ostrich TLR4 was closely related with ancient species, which indicated that ostrich could be a good model for evolutionary analysis in birds. The evolutionary analysis of full-length TLR4 in 37 birds showed that purifying selection occurred across all TLR4 protein, and positive selection on some codons, which is the same as the previous study [27]. Moreover, positive selection was observed in non-ECD domains (TM and ICD), which was not mentioned in previous studies. In addition, more positively selected sites were found in ECD (72 sites vs. 24 sites) than before, which could be a supplement for the studies about TLR4 evolution in birds [27]. 84~92% mutated sites under positive selection were located in ECD. Since ECD is functionally important for ligand binding, the high probabilities of positive selection in this region, may suggest a ligand-mediated adoptive selection for TLR4 in avian species as discussed in previous studies [27].

Both the transcripts of TLR3 and TLR4 were detected with different levels in ostrich thymus and bursa of fabricius, the central immune organ for the development of T cells and B cells respectively. TLR3 was preferentially expressed in bursa of fabricius, whereas TLR4 expressed in ostrich thymus. Their differential expression pattern in primary immune organs may reflect the diverse demands of TLRs in these organs. Using tested antibody against avian TLR4, we found that TLR4 was distributed in specific regions of both thymus and bursa of fabricius. In thymus, TLR4 was mainly observed in medulla and cortico-medullary junction, the later developing region for thymocytes [34]. In bursa of fabricius, TLR4 was mainly located in mucosal epithelium, the part that may directly engage the antigen pulled into bursal lumen [35], and in medulla, the potential region for the selection of longer lived population of B cells by antigen [36]. Furthermore, TLR4 was located in thymic PCK + epithelial cells and S100 + dendritic cells, but not eosinophils. Therefore, the strategic distribution of TLR4 in ostrich thymus may reflect possible participation in thymic development, by initiating immune reaction in thymic epithelial cells and dendritic cells.

Furthermore, in normal thymus, TLR4 expression increased significantly, and TLR3 expression showed an increased tendency with age, similar to what had been described in bird and mammals [16, 17, 37]. This may be related to the increased demands of intrathymic immune surveillance with age. The protection from maternal antibodies fades away gradually during postnatal development [38]. The consistent expression of TLR downstream products, including transcriptional regulators (IRF1, p100, JNK, ERK and p38) and proinflammatory cytokines (IFN and IL6), in ostrich thymus may contribute to intrathymic homeostasis during normal development, since abnormal expression of them are associated with aberrant thymic development [18–21].

In boron-stress thymus of ostriches, TLR4 expression decreased significantly and TLR3 expression decreased modestly compared to those in normal developing thymus at day 90. There is no change in the proinflammatory transcriptional regulators (IRF1, JNK, ERK and p38) and proinflammatory cytokines (IFN and IL6) following boron stress. However, the transcriptional regulator (p100) increased. Impaired expression TLRs are associated with inhibition of TLRs function [39, 40], and p100 can inhibit NF-κB activity by binding the active elements of NF-κB [41]. Therefore, although TLRs may control cell survival by downstream factors [42], there were no increasing demands of TLRs and downstream proinflammatory factors in thymus under boron stress. In addition, inhibition of TLRs pathway (e.g., TLR4, JNK, IFN-α/β or IFN-γ) could not promote cell proliferation or apoptosis during pathogenic stress [43–46]. Thus, increased thymic apoptosis, autophagy and proliferation after long-term intake of excessive boron may not associate with the TLRs pathway. Besides, inhibition of TLRs function under boron stress may respond to the decreased inflammatory response to pathogens under boron stress [47, 48].

In conclusion, long-term excessive boron intake did not change ostrich thymic organization. The resistance of ostrich thymus against long-term boron stress may rely on the accelerating intrathymic cell renew and death, which may bypass TLR3/4 pathway, since TLR3 and TLR4 expression decreased after boron treatment. The decreased TLRs activity may explain the immune suppression under boron stress in other studies. Yet, the precise mechanisms for regulating thymic cell fate and immune response under boron stress deserve further investigation.

## Supporting Information

S1 File Colocation of TLR4 and stromal cells in ostrich thymus. The TLR4-positive signals (**Figure A**) and PCK-positive signals (**Figure B**) were found in the similar regions in adjacent sections of ostrich thymus at day 90. TLR4-positive signals (**Figure C**) and S100-positive signals (**Figure D**) were also found in the similar regions in adjacent sections of ostrich thymus at day 45. However, TLR4-positvie signals were not observed in eosinophils in ostrich thymus (**Figure E**). The regions marked by black arrows in **Figure E** are enlarged in right corner of this panel. Bars, 100 μm.
(TIF)

S1 Table The antibodies used in this study. The identities between the targeted antigen sequences of those antibodies and those found in ostrich were compared using NCBI BLAST program.
(DOC)

S2 Table The references sequences used to design degenerate primers. Sequences XM_003211211 (TLR4) and XM_003205774 (TLR3) for *Meleagris gallopavo* were updated in December 2014.
(DOC)

S3 Table The 36 newly registered birds genomes in NCBI by BGI. For adaptive evolution analysis, we adopted 37 TLR4 mRNA sequences from birds, including the 30 known ones, except that from ostrich, listed in this table, the 6 ones listed in S4 Table, and the ostrich TLR4 mRNA (KM408431) from our cloning results.
(DOC)

S4 Table The retrieved TLR4 sequences of reptiles, mammals, fish and other birds from NCBI. For compared genomic structure of TLR4 genes, we used 6 genomic sequences of TLR4, including 5 ones from zebrafish, human, chinese soft-shell turtle, chicken and mallard listed in this table, and one from ostrich, which was deduced from our cloning TLR4 mRNA (KM408431) and ostrich genomic scaffold (KL205999.1) provided by BGI. For phylogenetic analysis, we adopted 49 TLR4 protein sequences from different species, including the 13 ones listed in this table, the 35 ones except that of ostrich listed in S3 Table and the ostrich TLR4 protein deduced from our cloning result (KM408431).
(DOC)
