# Supplementary figures and images for: Increased Thymic Cell Turnover under Boron Stress May Bypass TLR3/4 Pathway in African Ostrich
Source: PLoS One. 2015 Jun 8;10(6):e0129596. doi: 10.1371/journal.pone.0129596 (PMC4460079; doi:10.1371/journal.pone.0129596)

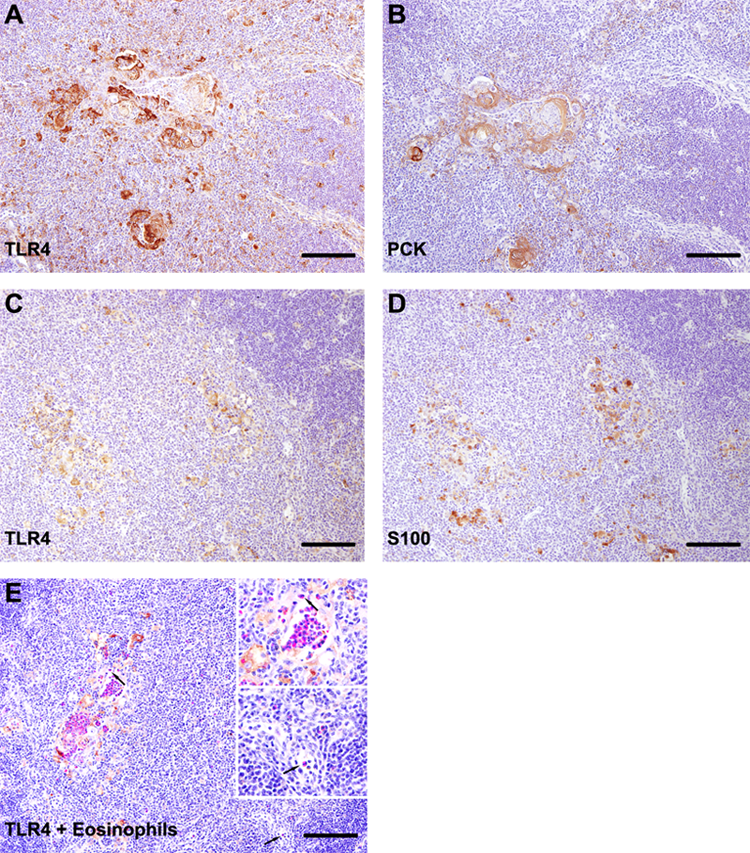

Supplement: S1 File — The TLR4-positive signals (Figure A) and PCK-positive signals (Figure B) were found in the similar regions in adjacent sections of ostrich thymus at day 90. TLR4-positive signals (Figure C) and S100-positive signals (Figure D) were also found in the similar regions in adjacent sections of ostrich thymus at day 45. However, TLR4-positvie signals were not observed in eosinophils in ostrich thymus (Figure E). The regions marked by black arrows in Figure E are enlarged in right corner of this panel. Bars, 100 μm. (TIF) [file pone.0129596.s001.tif]
