# Supplementary material for: Increased Thymic Cell Turnover under Boron Stress May Bypass TLR3/4 Pathway in African Ostrich
Source: PLoS One. 2015 Jun 8;10(6):e0129596. doi: 10.1371/journal.pone.0129596 (PMC4460079; doi:10.1371/journal.pone.0129596)
Supplement: S1 Table — The identities between the targeted antigen sequences of those antibodies and those found in ostrich were compared using NCBI BLAST program. (DOC) [file pone.0129596.s002.doc]

**S1 Table. The antibodies used in this study.**

| **Antibody** | **Species specificity** | **Source** | **Catalog NO.** |
| --- | --- | --- | --- |
| Anti-TLR4 (rabbit, IgG) | Human (73% identity with ostrich) | Boster | BA1717 |
| Anti-LC3A/B (rabbit, IgG) | Avian | Abcom | ab58610 |
| Anti-PCNA (mouse, IgG_2a_) | Rat (97% identity with ostrich) | Santa Cruz | sc-56 |
| Anti-PCK (PCK-26,mouse, IgG1) | Avian | Abcom | ab6401 |
| Anti-S100 (rabbit, IgG) | Avian | Abcom | ab74161 |
| Anti-ssDNA (mouse, IgM) | Specific for ssDNA | Chemicon | MAB3299 |
| Anti-JNK (mouse, IgG1) | Human (81% identity with ostrich) | Santa Cruz | sc7345 |
| Anti-ERK (rabbit, IgG)) | Human (80% identity with ostrich) | Bioworld | BS1112 |
| Anti-P38 (rabbit, IgG) | Human (96% identity with ostrich) | Epitomics | 1544-1 |
| Actin (mouse, IgG) | Avian | Santa cruz | sc-1616r |

The identities between the targeted antigen sequences of those antibodies and those found in ostrich were compared using NCBI BLAST program.
