# Supplementary material for: Increased Thymic Cell Turnover under Boron Stress May Bypass TLR3/4 Pathway in African Ostrich
Source: PLoS One. 2015 Jun 8;10(6):e0129596. doi: 10.1371/journal.pone.0129596 (PMC4460079; doi:10.1371/journal.pone.0129596)
Supplement: S2 Table — Sequences XM_003211211 (TLR4) and XM_003205774 (TLR3) for Meleagris gallopavo were updated in December 2014. (DOC) [file pone.0129596.s003.doc]

**S2** **Table. The references sequences used to design degenerate primers.**

| **Target gene** | **References sequences** | **Organism** | **Registration date** |
| --- | --- | --- | --- |
| TLR4 | NM_001030693 | Gallus gallus | 2014-3 |
|  | JQ713173 | Perdix perdix | 2012-8 |
|  | XM_003211211 | Meleagris gallopavo | 2011-3 |
|  | HQ436371 | Anser anser | 2011-5 |
|  | XM_005025640 | Anas platyrhynchos | 2013-7 |
|  | XM_005441990 | Falco cherrug | 2013-9 |
|  | XM_005145616 | Melopsittacus undulatus | 2013-6 |
|  | XM_005498384 | Columba livia | 2013-8 |
|  | XM_005527197 | Pseudopodoces humilis | 2013-9 |
|  | FJ695612 | Taeniopygia guttata | 2009-7 |
|  | XM_005423810 | Geospiza fortis | 2013-9 |
|  |  |  |  |
| TLR3 | XM_003205774 | Meleagris gallopavo | 2013-3 |
|  | XM_005008981 | Anas platyrhynchos | 2013-7 |
|  | XM_005500210 | Columba livia | 2013-9 |
|  | KC292270 | Anser anser | 2013-7 |
|  | XM_005243141 | Falco peregrinus | 2013-8 |
|  | XM_005433774 | Falco cherrug | 2013-9 |
|  | XM_005149053 | Melopsittacus undulatus | 2013-7 |
|  | XM_002190852 | Taeniopygia guttata | 2013-2 |
|  | XM_005518005 | Pseudopodoces humilis | 2013-9 |
|  | XM_005415516 | Geospiza fortis | 2013-9 |
|  | XM_005483864 | Zonotrichia albicollis | 2013-9 |
|  | XM_006260649 | Alligator mississippiensis | 2013-11 |

Sequences XM_003211211 (TLR4) and XM_003205774 (TLR3) for *Meleagris gallopavo* were updated in December 2014.
