# Supplementary material for: Increased Thymic Cell Turnover under Boron Stress May Bypass TLR3/4 Pathway in African Ostrich
Source: PLoS One. 2015 Jun 8;10(6):e0129596. doi: 10.1371/journal.pone.0129596 (PMC4460079; doi:10.1371/journal.pone.0129596)
Supplement: S3 Table — For adaptive evolution analysis, we adopted 37 TLR4 mRNA sequences from birds, including the 30 known ones, except that from ostrich, listed in this table, the 6 ones listed in S4 Table, and the ostrich TLR4 mRNA (KM408431) from our cloning results. (DOC) [file pone.0129596.s004.doc]

**S3 Table. The 36 newly registered birds genomes in NCBI by BGI.**

| **BioProject** | **Organism** | **Registration date** | | **TLR4 mRNA** | **TLR4 protein** |
| --- | --- | --- | --- | --- | --- |
| 265998 | Phaethon lepturus (white-tailed tropicbird) | | 2014-4-30 | － | KFQ81324.1 |
| 212904 | Phoenicopterus ruber ruber | | 2014-4-30 | － | KFQ89334.1 |
| 263629 | Tyto alba (barn owl) | | 2014-4-30 | [XM_009970420.1](http://www.ncbi.nlm.nih.gov/nuccore/XM_009970420.1) | [XP_009968722.1](http://www.ncbi.nlm.nih.gov/protein/XP_009968722.1) |
| 253837 | Merops nubicus (carmine bee-eater) | | 2014-5-1 | [XM_008945045.1](http://www.ncbi.nlm.nih.gov/nuccore/XM_008945045.1) | [XP_008943293.1](http://www.ncbi.nlm.nih.gov/protein/XP_008943293.1) |
| 265381 | Mesitornis unicolor (brown roatelo) | | 2014-5-1 | [XM_010193770.1](http://www.ncbi.nlm.nih.gov/nuccore/XM_010193770.1) | [XP_010192072.1](http://www.ncbi.nlm.nih.gov/protein/XP_010192072.1) |
| 264990 | Nestor notabilis (Kea) | | 2014-5-1 | [XM_010014853.1](http://www.ncbi.nlm.nih.gov/nuccore/XM_010014853.1) | [XP_010013155.1](http://www.ncbi.nlm.nih.gov/protein/XP_010013155.1) |
| 253833 | Pelecanus crispus (Dalmatian pelican) | | 2014-5-1 | [XM_009481688.1](http://www.ncbi.nlm.nih.gov/nuccore/XM_009481688.1) | [XP_009479963.1](http://www.ncbi.nlm.nih.gov/protein/XP_009479963.1) |
| 263514 | Haliaeetus albicilla (white-tailed eagle) | | 2014-5-2 | [XM_009915776.1](http://www.ncbi.nlm.nih.gov/nuccore/XM_009915776.1) | [XP_009914078.1](http://www.ncbi.nlm.nih.gov/protein/XP_009914078.1) |
| 263616 | Leptosomus discolor (cuckoo roller) | | 2014-5-2 | [XM_009947763.1](http://www.ncbi.nlm.nih.gov/nuccore/XM_009947763.1) | [XP_009946065.1](http://www.ncbi.nlm.nih.gov/protein/XP_009946065.1) |
| 261828 | Fulmarus glacialis (northern fulmar) | | 2014-5-3 | [XM_009586474.1](http://www.ncbi.nlm.nih.gov/nuccore/XM_009586474.1) | [XP_009584769.1](http://www.ncbi.nlm.nih.gov/protein/XP_009584769.1) |
| 261843 | Gavia stellata (red-throated loon) | | 2014-5-3 | [XM_009809127.1](http://www.ncbi.nlm.nih.gov/nuccore/XM_009809127.1) | [XP_009807429.1](http://www.ncbi.nlm.nih.gov/protein/XP_009807429.1) |
| 263623 | Cariama cristata (red-legged seriema) | | 2014-5-5 | [XM_009702452.1](http://www.ncbi.nlm.nih.gov/nuccore/XM_009702452.1) | [XP_009700754.1](http://www.ncbi.nlm.nih.gov/protein/XP_009700754.1) |
| 265879 | Colius striatus (speckled mousebird) | | 2014-5-5 | [XM_010195927.1](http://www.ncbi.nlm.nih.gov/nuccore/XM_010195927.1) | [XP_010194229.1](http://www.ncbi.nlm.nih.gov/protein/XP_010194229.1) |
| 265393  253841 | Eurypyga helias (sunbittern)  Acanthisitta chloris (rifleman) | | 2014-5-5  2014-5-6 | [XM_010156250.1](http://www.ncbi.nlm.nih.gov/nuccore/XM_010156250.1)  [XM_009073130.1](http://www.ncbi.nlm.nih.gov/nuccore/XM_009073130.1) | [XP_010154552.1](http://www.ncbi.nlm.nih.gov/protein/XP_010154552.1)  [XP_009071378.1](http://www.ncbi.nlm.nih.gov/protein/XP_009071378.1) |
| 253828 | Calypte anna (Anna's hummingbird) | | 2014-5-6 | [XM_008499333.1](http://www.ncbi.nlm.nih.gov/nuccore/XM_008499333.1) | [XP_008497555.1](http://www.ncbi.nlm.nih.gov/protein/XP_008497555.1) |
| 263502 | Picoides pubescens (downy woodpecker) | | 2014-5-6 | [XM_009907081.1](http://www.ncbi.nlm.nih.gov/nuccore/XM_009907081.1) | [XP_009905383.1](http://www.ncbi.nlm.nih.gov/protein/XP_009905383.1) |
| **263340** | **Struthio camelus australis (African ostrich)** | | **2014-5-6** | [**XM_009668178.1**](http://www.ncbi.nlm.nih.gov/nuccore/XM_009668178.1) | [**XP_009666473.1**](http://www.ncbi.nlm.nih.gov/protein/XP_009666473.1) |
| 261081 | Aptenodytes forsteri (emperor penguin) | | 2014-5-7 | [XM_009282256.1](http://www.ncbi.nlm.nih.gov/nuccore/XM_009282256.1) | [XP_009280531.1](http://www.ncbi.nlm.nih.gov/protein/XP_009280531.1) |
| 212905 | Podiceps cristatus (great crested grebe) | | 2014-5-7 | － | KFZ49176.1 |
| 265368 | Pterocles gutturalis (yellow-throated sandgrouse) | | 2014-5-7 | [XM_010073689.1](http://www.ncbi.nlm.nih.gov/nuccore/XM_010073689.1) | [XP_010071991.1](http://www.ncbi.nlm.nih.gov/protein/XP_010071991.1) |
| 261076 | Pygoscelis adeliae (Adelie penguin) | | 2014-5-7 | [XM_009319316.1](http://www.ncbi.nlm.nih.gov/nuccore/XM_009319316.1) | [XP_009317591.1](http://www.ncbi.nlm.nih.gov/protein/XP_009317591.1) |
| 263608 | Apaloderma vittatum (bar-tailed trogon) | | 2014-5-8 | [XM_009867818.1](http://www.ncbi.nlm.nih.gov/nuccore/XM_009867818.1) | [XP_009866120.1](http://www.ncbi.nlm.nih.gov/protein/XP_009866120.1) |
| 265383 | Caprimulgus carolinensis (chuck-will's-widow) | | 2014-5-8 | [XM_010165071.1](http://www.ncbi.nlm.nih.gov/nuccore/XM_010165071.1) | [XP_010163373.1](http://www.ncbi.nlm.nih.gov/protein/XP_010163373.1) |
| 265201 | Tinamus guttatus (white-throated tinamou) | | 2014-5-8 | － | KGL80424 |
| 263452 | Charadrius vociferus (killdeer) | | 2014-5-9 | [XM_009888044.1](http://www.ncbi.nlm.nih.gov/nuccore/XM_009888044.1) | [XP_009886346.1](http://www.ncbi.nlm.nih.gov/protein/XP_009886346.1) |
| 266006 | Chlamydotis macqueenii (Macqueen's bustard) | | 2014-5-12 | [XM_010127205.1](http://www.ncbi.nlm.nih.gov/nuccore/XM_010127205.1) | [XP_010125507.1](http://www.ncbi.nlm.nih.gov/protein/XP_010125507.1) |
| 261839 | Phalacrocorax carbo (great cormorant) | | 2014-5-12 | [XM_009514274.1](http://www.ncbi.nlm.nih.gov/nuccore/XM_009514274.1) | [XP_009512569.1](http://www.ncbi.nlm.nih.gov/protein/XP_009512569.1) |
| 253849 | Corvus brachyrhynchos (American crow) | | 2014-5-13 | [XM_008638328.1](http://www.ncbi.nlm.nih.gov/nuccore/XM_008638328.1) | [XP_008636550.1](http://www.ncbi.nlm.nih.gov/protein/XP_008636550.1) |
| 253835 | Manacus vitellinus (golden-collared manakin) | | 2014-5-13 | [XM_008920440.1](http://www.ncbi.nlm.nih.gov/nuccore/XM_008920440.1) | [XP_008918688.1](http://www.ncbi.nlm.nih.gov/protein/XP_008918688.1) |
| 263612 | Opisthocomus hoazin | | 2014-5-13 | [XM_009935409.1](http://www.ncbi.nlm.nih.gov/nuccore/XM_009935409.1) | [XP_009933711.1](http://www.ncbi.nlm.nih.gov/protein/XP_009933711.1) |
| 264996 | Balearica regulorum gibbericeps | | 2014-6-2 | － | KFO11637.1 |
| 266010 | Buceros rhinoceros silvestris | | 2014-6-2 | [XM_010137451.1](http://www.ncbi.nlm.nih.gov/nuccore/XM_010137451.1) | [XP_010135753.1](http://www.ncbi.nlm.nih.gov/protein/XP_010135753.1) |
| 263299 | Cuculus canorus (common cuckoo) | | 2014-6-12 | [XM_009569425.1](http://www.ncbi.nlm.nih.gov/nuccore/XM_009569425.1) | [XP_009567720.1](http://www.ncbi.nlm.nih.gov/protein/XP_009567720.1) |
| 265115 | Tauraco erythrolophus (red-crested turaco) | | 2014-6-12 | [XM_009978945.1](http://www.ncbi.nlm.nih.gov/nuccore/XM_009978945.1) | [XP_009977247.1](http://www.ncbi.nlm.nih.gov/protein/XP_009977247.1) |
| 264987 | Chaetura pelagica (chimney swift) | | 2014-9-4 | [XM_010007952.1](http://www.ncbi.nlm.nih.gov/nuccore/XM_010007952.1) | [XP_010006254.1](http://www.ncbi.nlm.nih.gov/protein/XP_010006254.1) |

For adaptive evolution analysis, we adopted 37 TLR4 mRNA sequences from birds, including the 30 known ones, except that from ostrich, listed in this table, the 6 ones listed in S4 Table, and the ostrich TLR4 mRNA (KM408431) from our cloning results.
