# Supplementary material for: Increased Thymic Cell Turnover under Boron Stress May Bypass TLR3/4 Pathway in African Ostrich
Source: PLoS One. 2015 Jun 8;10(6):e0129596. doi: 10.1371/journal.pone.0129596 (PMC4460079; doi:10.1371/journal.pone.0129596)
Supplement: S4 Table — For compared genomic structure of TLR4 genes, we used 6 genomic sequences of TLR4, including 5 ones from zebrafish, human, chinese soft-shell turtle, chicken and mallard listed in this table, and one from ostrich, which was deduced from our cloning TLR4 mRNA (KM408431) and ostrich genomic scaffold (KL205999.1) provided by BGI. For phylogenetic analysis, we adopted 49 TLR4 protein sequences from different species, including the 13 ones listed in this table, the 35 ones except that of ostrich listed in S3 Table and the ostrich TLR4 protein deduced from our cloning result (KM408431). (DOC) [file pone.0129596.s005.doc]

**S4 Table. The retrieved TLR4 sequences** **of reptiles, mammals, fish and other birds from NCBI.**

| **Taxonomy** | **Organism** | **Gene** | **mRNA** | **Protein** |
| --- | --- | --- | --- | --- |
| Mammals | Homo sapiens (human) | NG_011475.1 | NM_138554.4 | NP_612564.1 |
|  | Bos taurus (cattle) | NC_007306.5 | NM_174198.6 | NP_776623.5 |
| Reptiles | Chelonia mydas (green turtle)) | NW_006622076.1 | XM_007057276.1 | XP_007057338.1 |
|  | Pelodiscus sinensis (chinese soft-shell turtle) | NW_005854608.1 | NM_001286933.1 | NP_001273862.1 |
|  | Anolis carolinensis (green anole) | NW_003338997.1 | XM_008120209.1 | XP_008118416.1 |
| Fish | Danio rerio (zebrafish) | NC_007124.6 | NM_212813.1 | NP_997978.1 |
|  | Ctenopharyngodon idella (grass carp) | JF965435.1 | － | AEQ64880.1 |
| Birds | Gallus gallus (chicken) | NC_006104.3 | NM_001030693.1 | NP_001025864.1 |
|  | Columba livia (rock pigeon) | NW_004973184.1 | XM_005498384.1 | XP_005498441.1 |
|  | Falco cherrug (Saker falcon) | NW_004994811.1 | XM_005441990.1 | XP_005442047.1 |
|  | Falco peregrinus (peregrine falcon) | NW_004929794.1 | XM_005231393.1 | XP_005231450.1 |
|  | Anas platyrhynchos (mallard) | NW_004677730.1 | XM_005025640.1 | XP_005025697.1 |
|  | Geospiza fortis (medium ground-finch) | NW_005054358.1 | XM_005423810.1 | XP_005423867.1 |

For compared genomic structure of TLR4 genes, we used 6 genomic sequences of TLR4, including 5 ones from zebrafish, human, chinese soft-shell turtle, chicken and mallard listed in this table, and one from ostrich, which was deduced from our cloning TLR4 mRNA (KM408431) and ostrich genomic scaffold (KL205999.1) provided by BGI.

For phylogenetic analysis, we adopted 49 TLR4 protein sequences from different species, including the 13 ones listed in this table, the 35 ones except that of ostrich listed in S3 Table and the ostrich TLR4 protein deduced from our cloning result (KM408431).
